# Treatment of secondary CNS lymphoma using CD19-targeted chimeric antigen receptor (CAR) T cells

**DOI:** 10.1007/s00262-023-03619-9

**Published:** 2024-02-13

**Authors:** Kathryn Kline, Tim Luetkens, Rima Koka, Michael E. Kallen, Wengen Chen, Haroon Ahmad, Destiny Omili, Thierry Iraguha, Etse Gebru, Xiaoxuan Fan, Alexis Miller, Nishanthini Dishanthan, Jillian M. Baker, Kenneth A. Dietze, Kim G. Hankey, Jean A. Yared, Nancy M. Hardy, Aaron P. Rapoport, Saurabh Dahiya, Djordje Atanackovic

**Affiliations:** 1 Cancer Immunotherapy, Fannie Angelos Cellular Therapeutics GMP Laboratory, University of Maryland Greenebaum Comprehensive Cancer Center, Bressler Research Building, Room 9-011, 655 W. Baltimore Street, Baltimore, MD 21201 USA; 2 Department of Medicine, University of Maryland School of Medicine, Baltimore, MD USA; 3 Department of Microbiology and Immunology, University of Maryland, Baltimore, MD USA; 4 Department of Pathology, University of Maryland School of Medicine, Baltimore, MD USA; 5 Diagnostic Radiology and Nuclear Medicine, University of Maryland School of Medicine, Baltimore, MD USA; 6 Department of Neurology, University of Maryland School of Medicine, Baltimore, MD USA; 7 Transplant and Cellular Therapy Program, University of Maryland Greenebaum Comprehensive Cancer Center, Baltimore, MD USA; 8 Stanford University, Stanford, CA USA

**Keywords:** B cell lymphoma, CNS lymphoma, CAR-T cells, Neurotoxicity, T cells, Cytokines

## Abstract

**Background:**

Aggressive B cell lymphoma with secondary central nervous system (CNS) involvement (SCNSL) carries a dismal prognosis. Chimeric antigen receptor (CAR) T cells (CAR-T) targeting CD19 have revolutionized the treatment for B cell lymphomas; however, only single cases with CNS manifestations successfully treated with CD19 CAR-T have been reported.

**Methods:**

We prospectively enrolled 4 patients with SCNSL into our study to assess clinical responses and monitor T cell immunity.

**Results:**

Two of four SNCSL patients responded to the CD19-targeted CAR-T. Only one patient showed a substantial expansion of peripheral (PB) CAR-T cells with an almost 100-fold increase within the first week after CAR-T. The same patient also showed marked neurotoxicity and progression of the SNCSL despite continuous surface expression of CD19 on the lymphoma cells and an accumulation of CD4^+^ central memory-type CAR-T cells in the CNS. Our studies indicate that the local production of chemokine IP-10, possibly through its receptor CXCR3 expressed on our patient’s CAR-T, could potentially have mediated the local accumulation of functionally suboptimal anti-tumor T cells.

**Conclusions:**

Our results demonstrate expansion and homing of CAR-T cells into the CNS in SNCSL patients. Local production of chemokines such as IP-10 may support CNS infiltration by CAR-T cells but also carry the potential of amplifying local toxicity. Future studies investigating numbers, phenotype, and function of CAR-T in the different body compartments of SNSCL patients receiving CAR-T will help to improve local delivery of “fit” and highly tumor-reactive CAR-T with low off-target reactivity into the CNS.

**Supplementary Information:**

The online version contains supplementary material available at 10.1007/s00262-023-03619-9.

## Introduction

Recurrent/relapsed aggressive B cell lymphoma with secondary central nervous system (CNS) involvement (SCNSL) carries a dismal prognosis. Complete remissions (CR) are rare, and responses are typically not durable [1]. Secondary CNS manifestations occur in 5% of patients with diffuse large B-cell lymphoma (DLBCL) with an overall survival (OS) of only 3–7 months [2, 3]. Historically, SCNSL patients have been excluded from clinical trials [4], highlighting the importance of reporting case series and conducting small-scale clinical trials to elucidate new therapeutic avenues. Chimeric antigen receptor (CAR) T cells (CAR-T) targeting CD19 have revolutionized the treatment for B cell lymphomas; however, only single cases of patients with CNS manifestations successfully treated with CD19 CAR-T have been reported [5]. We prospectively enrolled 4 patients with SCNSL into our study to assess clinical responses and perform a detailed assessment of pre- and post-treatment anti-tumor immunity.

## Materials and methods

### Patient samples

Blood and cerebrospinal fluid (CSF) samples were collected under Institutional Review Board (IRB)-approved protocol 2043GCCC (IRB HP-00091736). Plasma was generated by centrifugation at 400G and frozen immediately at − 80 °C. Peripheral blood mononuclear cells (PBMCs) were isolated using density gradient centrifugation and cryopreserved in liquid nitrogen.

### Immunohistochemistry (IHC)

Histologic sections from formalin-fixed, paraffin-embedded tissue samples underwent immunohistochemical and in situ hybridization staining using standard techniques.

### Flow cytometry

Prior to analysis of stained samples by flow cytometry, compensation settings were determined using single color controls and unstained cells. Single color controls were prepared using the MACS Comp Bead kit (Miltenyi #130-104-693). Cells were washed and stained in PBS containing 2% bovine serum albumin (BSA). Prior to flow-cytometric analysis of PBMC, staining was performed using a panel of monoclonal antibodies and CAR Detection Reagents (Supplementary Table 1) following manufacturer’s instructions. Costaining of cytoplasmic markers was performed following fixation and permeabilization, using Inside Fix and Inside Perm from the SARS-CoV-2 T Cell Analysis Kit (Miltenyi #130–128-034). Live cells were identified by 7-AAD dye exclusion (Miltenyi #130–111-568). Samples were acquired using a Miltenyi MACSQuant Analyzer 10 Flow Cytometer.

### CodePlex secretome analysis

Cytokine/chemokine concentrations in CSF and plasma samples were quantified using the CodePlex Secretome Human Adaptive Immune Panel kit (IsoPlexis # CODEPLEX-2L01). This panel measures the absolute concentration of 22 cytokines in a single sample using internal cytokine standards. To carry out the CodePlex analysis, chips were thawed at room temperature for 1 h, before supernatants were loaded onto the chip microchamber. The chip was then loaded into the Isolight reader (Isoplexis, Branfold, CT) and automated analysis of raw data was performed using IsoSpeak software (Isoplexis).

### Data analysis

Flow cytometry data were analyzed using FlowJo software version 10.9.0 (BD Biosciences, Franklin Lakes, NJ). Overall data analysis was performed using GraphPad Prism software version 9.5.1 (GraphPad Software, Boston, MA). Figures were composed using OmniGraffle software version 7.21.4 (The Omni Group, Seattle, WA).

## Results

Below we are providing a detailed description of the clinical course of our 4 SCNSL patients following i.v. infusion of CD19-targeted CAR-T cells:

Patient 1 was a 38-year-old man with Burkitt lymphoma with CNS progression who received whole brain radiation (WBR) shortly before lymphodepleting chemotherapy and lisocabtagene maraleucel (liso-cel) i.v. infusion (Table 1 and Supplemental Table 2). On day + 8 post CAR-T, he was noted to be more lethargic. Magnetic resonance imaging (MRI) of his brain showed progressive leptomeningeal and parenchymal disease. On day + 10 post CAR-T, he elected to be transitioned to hospice care.

**Table 1:** Patient characteristics

Patient	Patient 1	Patient 2	Patient 3	Patient 4
Age	38	48	34	33
Sex	Male	Male	Male	Male
Diagnosis	Burkitt	DLBCL	High-grade BCL	Burkitt
Stage	IV	IV	IV	IV
Type of CNS involvement	parenchymal	parenchymal	parenchymal	parenchymal

DLBCL = diffuse large B cell lymphoma; GCB = Germinal Center B cell-like

**Fig. 1:**
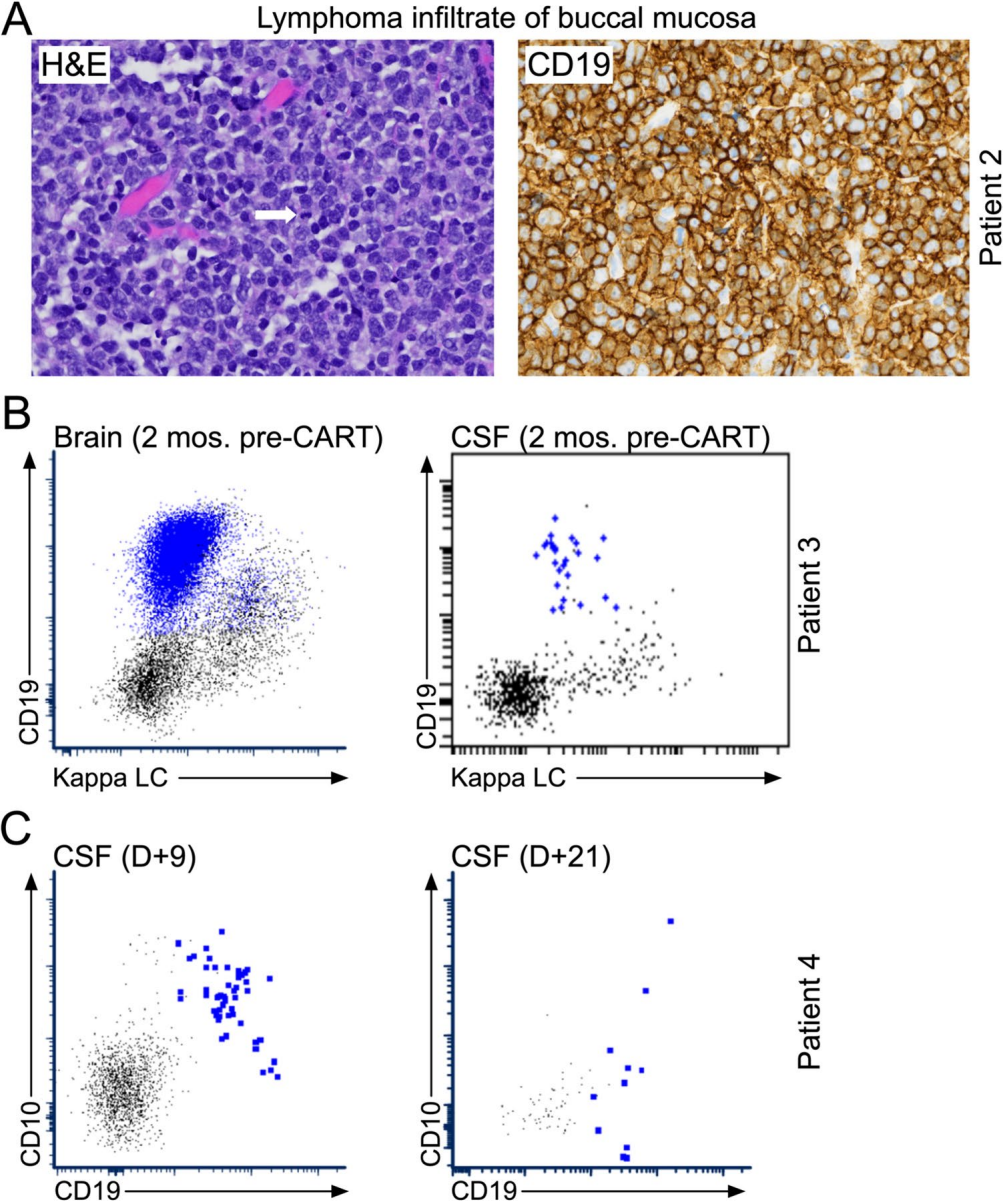
Antigen expression on tumor tissue. **A** Immunohistochemical analyses performed on tissue from the mandibular buccal mucosa of patient 2 at approximately 2 years prior to CAR-T cell therapy. Hematoxylin- and eosin-stained sections (left; 400 × magnification) are shown. Arrows indicate areas with large cells with frequent mitoses. The tumor cells were stained for expression of CD19 (right; 400 × magnification). **B** Analysis of CNS-infiltrating B cell lymphoma cells by flow cytometry in patient 3 at approximately two months prior to CAR-T cell therapy. The infiltrating tumor cells were stained for surface expression of CD19 antigen. **C** Analysis of CNS-infiltrating B cell lymphoma cells by flow cytometry in patient 4 at day + 9 (left) and day + 21 (right) after CAR-T cell therapy. Again, the infiltrating tumor cells were stained for surface expression of CD19 antigen

**Fig. 2:**
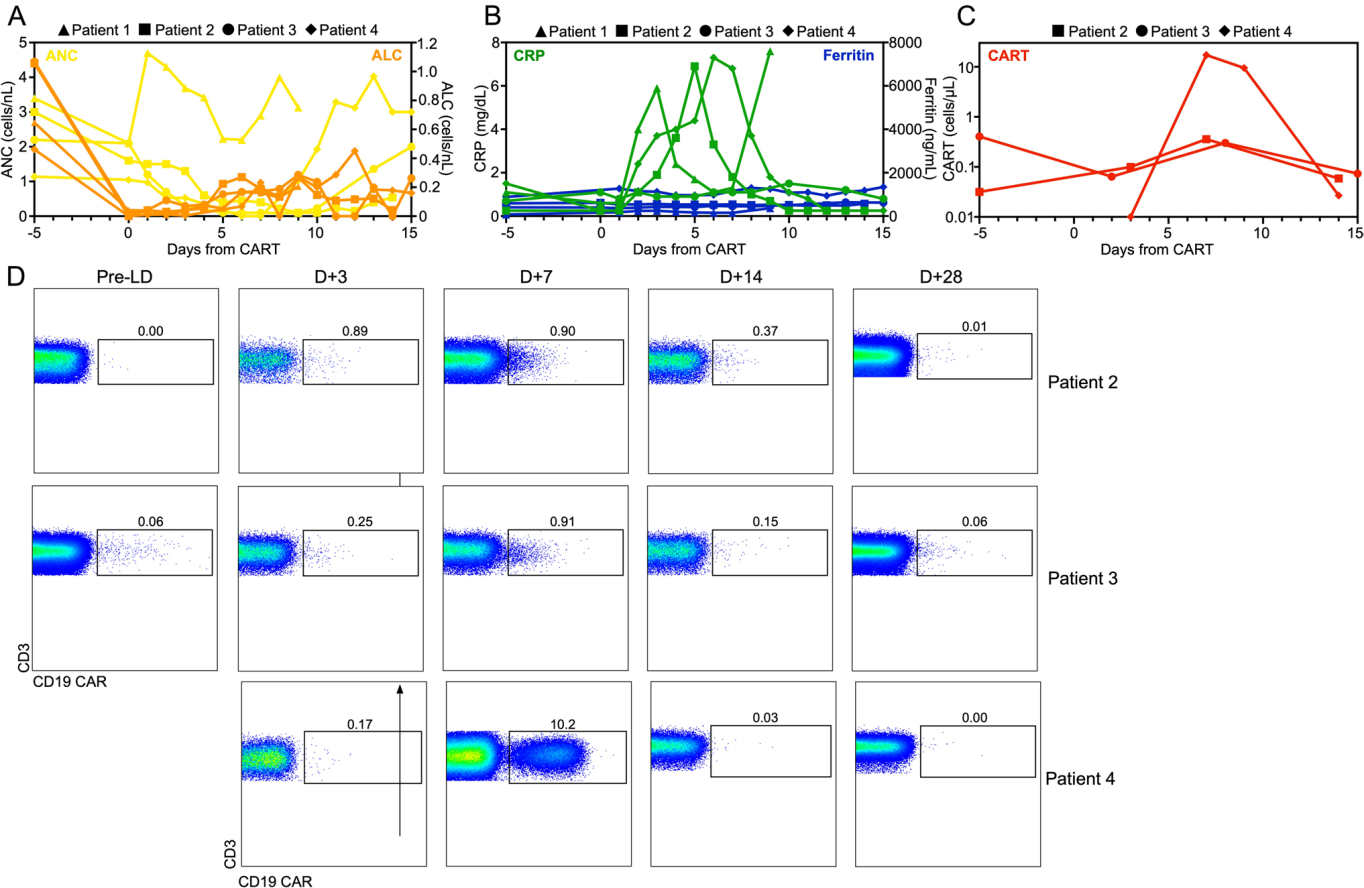
CAR-T cell expansion and persistence in patients with secondary CNS lymphoma post CD19 CAR-T cells. Time course of **A** reconstitution of absolute neutrophil counts (ANC) and absolute lymphocyte counts (ALC), **B** serum C-reactive protein (CRP) and ferritin levels, and **C** CAR-T cell numbers after lymphodepleting chemotherapy and CAR-T cell infusion. **D** Dot plots showing peripheral blood CAR-T cells in 3 of 4 patients at different timepoints post CAR-T infusion. CAR-T cells were identified by staining of the expression of the CAR on the cell surface and costaining with anti-CD3 and other T cell markers (Supplemental Table 1)

Patient 2 was a 48-year-old man with CD19-expressing (Fig. 1A) non-GCB (Germinal Center B-cell-like) DLBCL with CNS relapse. Prior to CAR-T he had received multiple lines of therapies (Table 1 and Supplemental Table 2). The dexamethasone used to manage brain edema was tapered prior to lymphodepletion and liso-cel infusion. He developed steroid-resistant grade 3 immune effector cell-associated neurotoxicity syndrome (ICANS) starting on day + 4 post CAR-T. He also developed hemophagocytic lymphohistiocytosis (HLH), with pancytopenia and elevated C-reactive protein (CRP), ferritin, and triglycerides (Fig. 2B) without improvement despite multiple doses of tocilizumab and siltuximab. An MRI of the brain on day + 30 indicated a partial remission (PR); however, his neurologic status continued to worsen. At day + 47, an MRI showed progression of his CNS manifestations. The patient was discharged to hospice care.

Patient 3 was a 32-year-old man with high-grade B-cell lymphoma (Table 1 and Supplemental Table 2) who had relapsed after multiple prior lines of therapy, including axi-cel given approximately 3 years earlier. At his third relapse with CD19-positive (Fig. 1B) CNS lesions, he received WBR followed by lymphodepletion and i.v. liso-cel. His initial post CAR-T course was uncomplicated, with no cytokine release syndrome (CRS) or ICANS. An MRI of the brain on day + 42 showed a decrease in the size of his brain lesions (Fig. 4A). Eight months after liso-cel, he had a systemic relapse of his lymphoma. He subsequently had a CR on a clinical trial of rituximab, gemcitabine, oxaliplatin, and zilovertamab vedotin and received an allogeneic stem cell transplant.

Patient 4 was a 33-year-old man with refractory Burkitt lymphoma with CNS involvement (Table 1 and Supplemental Table 2) receiving lymphodepletion and i.v. axi-cel. His post-infusion course was complicated by Grade 2 ICANS and multiple seizures. Lumbar puncture on day + 21 showed lymphoma cells in the cerebrospinal fluid (CSF) and an MRI of his brain on day + 26 showed new parenchymal lesions (Fig. 4B). Importantly, the CNS-infiltrating tumor cells at the time still expressed target antigen CD19 (Fig. 1C). His ICANS improved with steroids. He was discharged home to receive palliative radiation to his brain and died on day + 50.

In order to understand what had led to the heterogenous response pattern in our SCNSL patients, we performed a detailed analysis of CAR-T-mediated immune responses in their peripheral blood (PB) and CNS. Our patients showed a substantial decrease in their absolute lymphocyte and neutrophil counts after lymphodepleting (LD) chemotherapy followed by count recovery over the next 14 days (Fig. 2A). All patients, except patient 1, showed a transient increase in CRP levels in the week following CAR-T infusion with ferritin levels remaining low (Fig. 2B). Interestingly, patient 3 evidenced some pre-existing CD19-targeting CAR-T even at the pre-LD timepoint, probably based on his prior treatment with axi-cel (Fig. 2D). Out of 3 evaluable patients, only patient 4 showed a substantial expansion of absolute and relative numbers of PB CAR-T cells with an almost 100-fold increase within the first week after CAR-T (Fig. 2C and D), possibly based on the fact that the patient received CAR-T with the CD28 costimulatory domain leading to a more pronounced expansion.

**Fig. 3:**
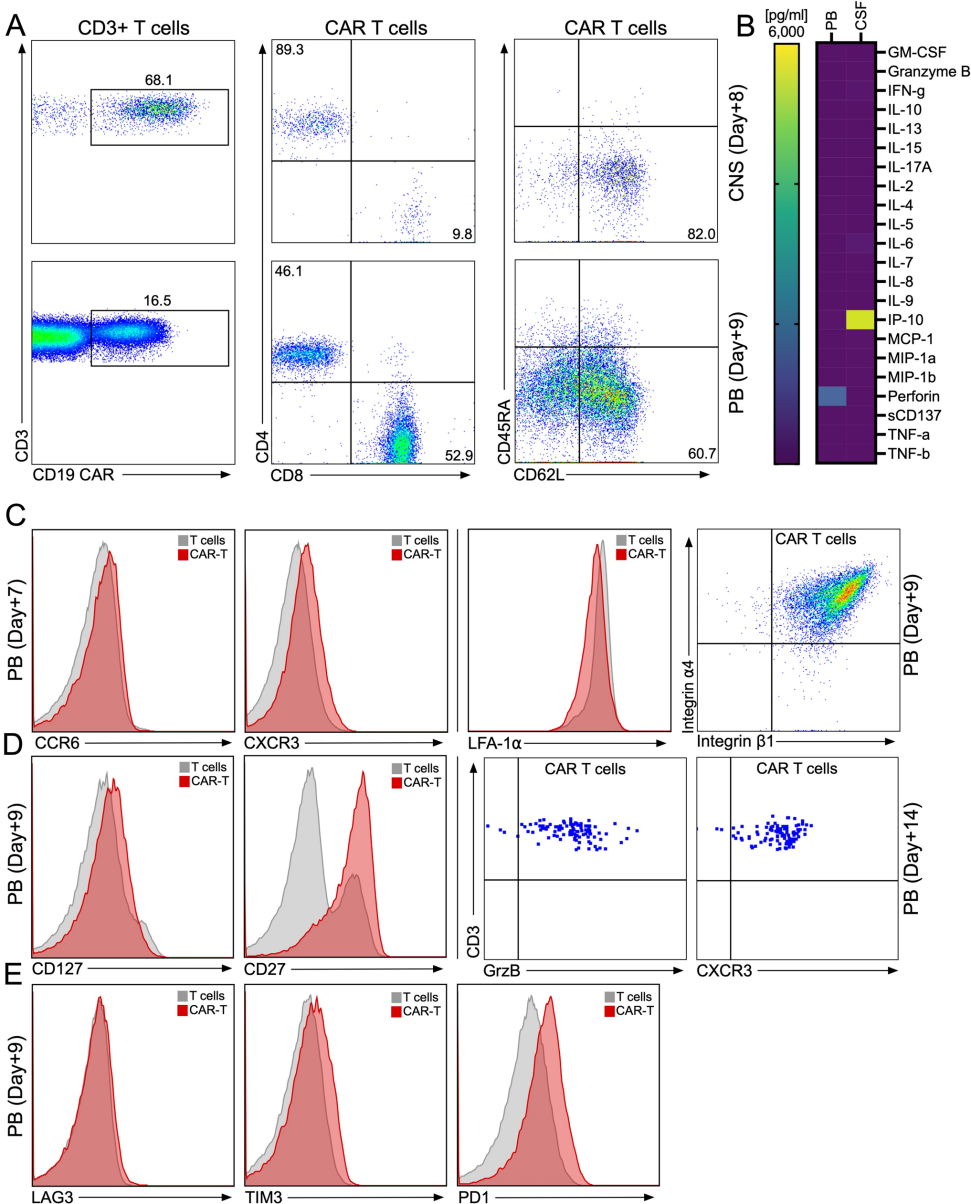
CNS infiltration by CD19-targeted CAR-T cells in a SCNSL patient with ICANS. **A** Proportions of CD4^+^ and CD8^+^ CAR-T cells were determined in the CSF (upper panel) and in the PB (lower panel) of patient 4 shortly after onset of potentially immune-mediated CNS toxicity using flow cytometry. Dot plots show CNS-infiltrating CAR-T cells at day + 8 (CSF) and day + 9 (PB), respectively. CAR-T cells were identified by staining of the expression of the CAR on the cell surface and costaining with anti-CD3, anti-CD4, anti-CD8, and other T cell markers (Supplemental Table 1). CAR-T cell memory subtypes were determined by costaining for CD45RA and CD62L. Central memory (CM) CAR-T cells are shown in the right lower quadrant. **B** Concentrations of 22 different T cell-related cytokines/chemokines were determined in our patient on days + 8 (left) and + 9 (right), respectively, at the onset of potentially immune-mediated CNS toxicity using CodePlex Secretome technology. Results are shown as absolute concentrations in pg/mL. **C** Histograms show surface expression of receptors involved in CNS-directed homing of T cells on PB CAR-T cells (red histograms) and non-CAR-T (gray histograms) from our patient measured on day + 7. The dot plot on the right shows proportions of peripheral blood CAR-T cells expressing α4β1 integrin required for the entry of T cells into the CNS. **D** Increased levels of CD27 and CD127 were found on PB CAR-T cells (red histograms) vs. non-CAR-T (gray histograms) as measured on day + 9. In addition, cytoplasmic granzyme B and surface levels of CXCR3 were determined on day + 14 post CAR-T cell treatment in PB CAR-T cells. **E** Expression of exhaustion markers on PB CAR-T cells (red histogram) from our patients compared to their own non-CAR-T (gray histogram) on day + 9 post CAR-T cell treatment

Trying to find an explanation for his severe neurologic symptoms following CAR-T infusion, we next analyzed the patient’s CSF for the presence of CAR-T. We found that his CSF indeed evidenced relatively high numbers of CD19-targeted CAR-T. In the CNS almost 70% of all T cells were CAR-T versus less than 20% in the PB (Fig. 3A). Furthermore, almost 90% of all CAR-T cells in the CNS were CD4-positive versus less than 50% in the PB (Fig. 3A). Finally, more than 80% of CAR-T in the CNS were central memory-type cells versus 60% in the periphery.

When we investigated which factors could have led to a recruitment of CAR-T into the CNS, we found that, unexpectedly, in this patient with the clinical picture of CNS inflammation, there was no increased local concentration of most of a total of 22 cytokines/chemokines analyzed in the CSF and PB. However, we observed a markedly increased concentration of chemokine IP10 (CXCL10) in the patient’s CSF versus his own PB (Fig. 3B). CXCR3 is a receptor for IP-10, and we found it to be overexpressed on the patient’s PB CAR-T versus their own non-CAR-T (Fig. 3C and D). Similarly, expression of CCR6, which is involved in recruiting activated T cells to the brain [6], was higher on the patient’s PB CAR-T cells compared to his non-CAR-T, whereas expression levels of lymphocyte function-associated antigen 1 (LFA-1) was similar in both groups (Fig. 3C). Finally, the patient’s CAR-T were uniformly positive for α4β1 integrin (Fig. 3C), a receptor required for T cell migration across the blood–brain barrier (BBB) [6, 7]. Apart from an overexpression of PD1, the patient’s PB CAR-T did not express any co-inhibitory molecules (Fig. 3E); however, they were CD127- [8] and granzyme B-positive, and they strongly overexpressed CD27 [9] (Fig. 3D), indicating full functionality.

## Discussion

From a clinical perspective, our results indicate a very heterogenous response pattern to CD19 CAR-T in patients with SCNSL. Moreover, in one of the two patients who responded, the response was only transient. This is consistent with two recent studies showing that even among SCNSL patients who respond to CAR-T, responses tend to only be temporary [10, 11]. Importantly, results look very similar for primary CNS lymphoma [12, 13]. While heterogenous response patterns could be related to the biology of the specific lymphoma subtype, in our study both of the patients with Burkitt lymphoma showed rapid progression after CAR-T, they could also be based on fundamental mechanisms inhibiting the clinical activity of CAR-T in CNS-infiltrating lymphomas.

**Fig. 4:**
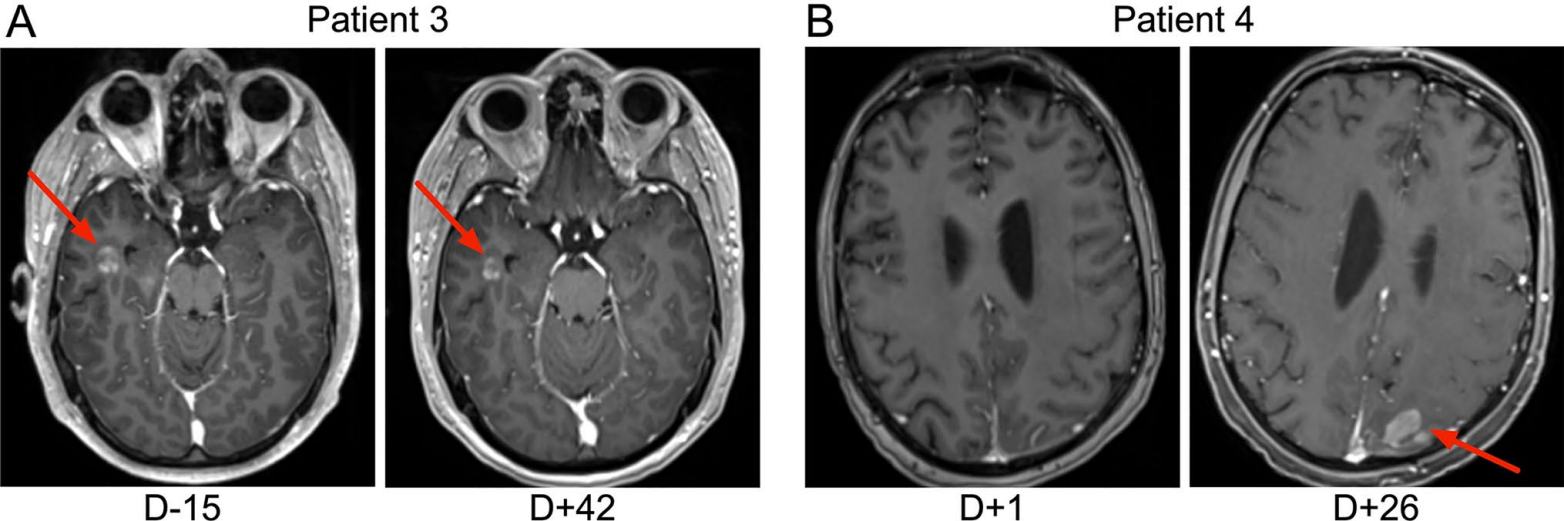
CNS imaging results post CD19 CAR-T cells. MRIs were taken on **A** patient 3 and **B** patient 4 at the timepoints indicated. In the case of patient 3, repeat imaging showed a decrease in the size of the brain lesion post CAR-T. For patient 4, imaging showed a new lesion in the left posterior parietal lobe

The CNS is protected by the BBB which potentially restricts local access of CAR-T to the CNS due to tight junctions maintained by endothelial cells [14]. Two recent murine studies of CNS lymphoma revealed that intravenous (i.v.) injection resulted in poor tumor infiltration of anti-CD19 CAR-T with insufficient control of tumor growth. In marked contrast, after intracerebral injection, anti-CD19 CAR-T cells invaded deeply into the solid tumor, reduced tumor growth, and induced regression of PCNSL, which was associated with long-term survival [15, 16]. Based on data like this, early phase clinical studies were initiated in different tumor types [17]. In a phase 1, clinical trial repetitive locoregional dosing of human erb-b2 receptor tyrosine kinase 2 (HER2)-specific CAR-T cells to children and young adults with recurrent/refractory CNS tumors, including diffuse midline glioma. Interestingly, patients treated with locally administered CAR-T developed high concentrations of IP-10 (CXCL10) and CCL2 in their cerebrospinal fluid [18] indicating local immune activation (Fig. 4).

Our data indicate that IP-10 could potentially play a role in the CNS recruitment of activated CAR-T, possibly through its receptor CXCR3 which has been studied extensively with regard to T cell recruitment during neuroinflammation. CXCR3 is abundantly expressed on CNS-infiltrating T cells in multiple sclerosis patients [19] and coordinates CNS-directed T cell migration in response to its three ligands, CXCL9/CXCL10/CXCL11 [20]. In addition to its potential role in orchestrating CNS-directed T cell migration, this chemokine and CXCR3 have also been shown to be involved in sensitivity to immune checkpoint inhibition and regulating T cell responses in general [21, 22]. Therefore, future studies should investigate in detail the role of IP-10/CXCR3 in promoting tumor-specific T cell responses, especially in patients with CNS lymphoma.

Our patient’s CAR-T also overexpressed CCR6 and interactions of CCR6 with its ligand CCL20 have been shown to be of importance for the attraction of effector T cells into the CNS [23, 24]. Furthermore, the patient’s CAR-T expressed α4β1 integrin which seems to be required for the entry of T cells into the CNS [25].

Our patient 4 did not respond to CD19 CAR-T treatment despite the fact that he had shown (1) a dramatic expansion of CAR-T in the periphery, (2) an accumulation of CD4^+^ central memory-type CAR-T cells in the CNS, and (3) continuous surface expression of CD19 on his CNS lymphoma cells. While infiltration of the CNS by the CAR-T cells is an absolute requirement for any anti-tumor activity to occur, our data also indicate that tumor tissue infiltration alone is not sufficient, at least not at the level observed in this study. It is important to keep in mind that, in addition to gaining highly restricted access to the CNS, tumor-specific T cells have to overcome an abundance of local immunosuppressive mechanisms leading to T cell senescence, exhaustion, and apoptosis [14]. In this context, we consider it possible that the CD4^+^ T cells that accumulated in our patient’s CNS could have represented regulatory-type T cells with a suboptimal effector function. Therefore, future studies should investigate how to further improve local delivery of CAR-T into the CNS and promote CNS infiltration by “fit” and highly tumor-reactive T cells, preferably with a low off-target reactivity.

### Supplementary Information

Below is the link to the electronic supplementary material.

Supplementary file1 (DOCX 21 KB)
