# Supplementary material for: Treatment of secondary CNS lymphoma using CD19-targeted chimeric antigen receptor (CAR) T cells
Source: Cancer Immunol Immunother. 2024 Feb 13;73(3):45. doi: 10.1007/s00262-023-03619-9 (PMC10864416; doi:10.1007/s00262-023-03619-9)
Supplement: Supplementary file 1 — Supplementary file1 (DOCX 21 KB) [file 262_2023_3619_MOESM1_ESM.docx]

**Supplemental Table 1: Monoclonal Antibodies for Flow Cytometry**

| **Target Antigen** | **Fluorophore** | **Manufacturer** | **CAT #** |
| --- | --- | --- | --- |
| Biotin | PE | Miltenyi | 130-110-951 |
| CAR Detection (BCMA) | Biotin | Miltenyi | 130-126-090 |
| CAR Detection (CD19) | Biotin | Miltenyi | 130-115-965 |
| CCR4 | APC | Miltenyi | 130-117-376 |
| CCR6 | PE-Vio770 | Miltenyi | 130-117-685 |
| CD3 | FITC | Miltenyi | 130-113-138 |
| CD3 | VioBlue | Miltenyi | 130-114-519 |
| CD4 | VioGreen | Miltenyi | 130-113-230 |
| CD8 | APC-Vio770 | Miltenyi | 130-110-681 |
| CD11a (LFA-1) | FITC | Miltenyi | 130-124-886 |
| CD27 | VioBlue | Miltenyi | 130-120-036 |
| CD29 (integrin β1) | APC | Miltenyi | 130-118-122 |
| CD45RA | VioBlue | Miltenyi | 130-117-743 |
| CD45RO | APC | Miltenyi | 130-113-556 |
| CD49d (integrin α4) | PE-Vio770 | Miltenyi | 130-125-506 |
| CD62L | PE-Vio770 | Miltenyi | 130-113-621 |
| CD127 | APC | Miltenyi | 130-113-413 |
| CD223 (LAG3) | VioBlue | Miltenyi | 130-118-549 |
| CD279 (PD1) | PE-Vio770 | Miltenyi | 130-120-385 |
| CD366 (TIM3) | APC | Miltenyi | 130-119-781 |
| CXCR3 | FITC | BioLegend | 353704 |
| Granzyme B | APC | Miltenyi | 130-120-703 |

**Supplemental Table 2: Treatments**

| **Patient:** | **Patient 1** | **Patient 2** | **Patient 3** | **Patient 4** |
| --- | --- | --- | --- | --- |
| Prior Treatments | 1. R-EPOCH 2. TEDD-R-copanlisib | 1. R-mini-CHOP 2. R-ICE 3. autoHCT with BEAM 4. TEDDi-R 5. VIPOR 6. Len/dex 7. WBR | 1. R-EPOCH 2. R-DHAP 3. TEDDI-R 4. WBR 5. axi-cel 6. GEMOX-ZV 7. alloSCT | 1. CODOX-M / IVAC 2. WBR 3. TEDD-R-copanlisib |
| Bridging | WBR | WBR, dexamethasone | None | None |
| Remission status at CAR-T | Active CNS disease | Active CNS disease | Complete remission | Active CNS disease |
| Type of CAR-T | Liso-cel | Liso-cel | Liso-cel | Axi-cel |
| Infused cells (10^6^/kg) | 50 x 10^6^ | 50 x 10^6^ | 5 x 10^6^ | Max of 2 x 10^8^ |
| CRS grade | None | None | None | None |
| Neurotoxicity | None | Grade 3 | None | Grade 2 |
| CRS/Neurotox treatment | None (dexamethasone for cerebral edema) | Dexamethasone, methylpred (max dose 1 g IV x 2) | None | Dexamethasone 10 mg IV q6hr 🡪 tapered |
| Best response | Progressive disease | Partial response | Partial response | Progressive disease |

WBR = Whole Brain Radiation; R-EPOCH = rituximab, etoposide, prednisone, vincristine, cyclophosphamide, doxorubicin; TEDD-R = temozolomide, etoposide, doxil, dexamethasone, rituximab; R-CHOP = rituximab, cyclophosphamide, doxorubicin, vincristine, prednisone; R-ICE = rituximab ifosfamide, carboplatin, etoposide; BEAM = carmustine (BCNU), etoposide, cytarabine, melphalan; TEDDi-R = temozolomide, etoposide, doxil, dexamethasone, ibrutinib, rituximab; VIPOR = venetoclax, ibrutinib, prednisone, obinutuzumab, and Revlimid (lenalidomide); Len = lenalidomide; R-DHAP = rituximab, dexamethasone, cytarabine, cisplatin; GEMOX = gemcitabine, oxaliplatin; ZV = Zilovertamab vedotin; allloSCT = allogeneic stem cell transplantation; R-CODOX-M = rituximab, cyclophosphamide, doxorubicin, vincristine, methotrexate; R-IVAC = rituximab, ifosfamide, cytarabine and etoposide; Axi-cel = axicabtagene ciloleucel; Liso-cel = lisocabtagene maraleucel
